# Comparative Analysis and Data Provenance for 1,113 Bacterial Genome Assemblies

**DOI:** 10.1128/msphere.00077-22

**Published:** 2022-05-02

**Authors:** David A. Yarmosh, Juan G. Lopera, Nikhita P. Puthuveetil, Patrick Ford Combs, Amy L. Reese, Corina Tabron, Amanda E. Pierola, James Duncan, Samuel R. Greenfield, Robert Marlow, Stephen King, Marco A. Riojas, John Bagnoli, Briana Benton, Jonathan L. Jacobs

**Affiliations:** a American Type Culture Collection (ATCC), Manassas, Virginia, USA; b BEI Resources, Manassas, Virginia, USA; University of Wisconsin—Madison

**Keywords:** DNA sequencing, bioinformatics, comparative studies, culture collection, data provenance, genome analysis, genome authentication, genomes, genomics, microbial genomics

## Abstract

The availability of public genomics data has become essential for modern life sciences research, yet the quality, traceability, and curation of these data have significant impacts on a broad range of microbial genomics research. While microbial genome databases such as NCBI’s RefSeq database leverage the scalability of crowd sourcing for growth, genomics data provenance and authenticity of the source materials used to produce data are not strict requirements. Here, we describe the *de novo* assembly of 1,113 bacterial genome references produced from authenticated materials sourced from the American Type Culture Collection (ATCC), each with full genomics data provenance relating to bioinformatics methods, quality control, and passage history. Comparative genomics analysis of ATCC standard reference genomes (ASRGs) revealed significant issues with regard to NCBI’s RefSeq bacterial genome assemblies related to completeness, mutations, structure, strain metadata, and gaps in traceability to the original biological source materials. Nearly half of RefSeq assemblies lack details on sample source information, sequencing technology, or bioinformatics methods. Deep curation of these records is not within the scope of NCBI’s core mission in supporting open science, which aims to collect sequence records that are submitted by the public. Nonetheless, we propose that gaps in metadata accuracy and data provenance represent an “elephant in the room” for microbial genomics research. Effectively addressing these issues will require raising the level of accountability for data depositors and acknowledging the need for higher expectations of quality among the researchers whose research depends on accurate and attributable reference genome data.

**IMPORTANCE** The traceability of microbial genomics data to authenticated physical biological materials is not a requirement for depositing these data into public genome databases. This creates significant risks for the reliability and data provenance of these important genomics research resources, the impact of which is not well understood. We sought to investigate this by carrying out a comparative genomics study of 1,113 ATCC standard reference genomes (ASRGs) produced by ATCC from authenticated and traceable materials using the latest sequencing technologies. We found widespread discrepancies in genome assembly quality, genetic variability, and the quality and completeness of the associated metadata among hundreds of reference genomes for ATCC strains found in NCBI’s RefSeq database. We present a comparative analysis of *de novo*-assembled ASRGs, their respective metadata, and variant analysis using RefSeq genomes as a reference. Although assembly quality in RefSeq has generally improved over time, we found that significant quality issues remain, especially as related to genomic data and metadata provenance. Our work highlights the importance of data authentication and provenance for the microbial genomics community, and underscores the risks of ignoring this issue in the future.

## INTRODUCTION

The National Center for Biotechnology Information’s (NCBI) RefSeq database has become an essential cornerstone of the global genomics research community, but the quality of metadata and the increasing need for manual data curation by end users are growing areas of concern (1–9). As RefSeq continues to expand, so too does the risk for data errors, omission, obfuscation, or falsification to go undetected, especially in large and aggregate bioinformatics analyses, and to potentially damage trust in this enormously important public resource (10, 11). RefSeq contains over 236,000 bacterial genome assemblies spanning more than 67,000 bacterial strains with assigned taxonomic identities. It is the largest collection of nonredundant, annotated genome assemblies available, and it is built exclusively from crowd-sourced data. However, despite extensive efforts to create automated curation pipelines and tools to improve RefSeq data, significant quality issues remain in genome assemblies found within RefSeq (12–14). For example, while all newly deposited prokaryote genome assemblies are automatically annotated, the associated metadata records (i.e., BioSample, BioProject, SRA, and Assembly data) are submitted by depositors who are not required to provide attribution for the biological materials behind each genome (8, 15). In fact, the International Nucleotide Sequence Database Collaboration (INSDC) policy states that “the quality and accuracy of the record are the responsibility of the submitting author, not of the database,” which is to say that metadata, which are often crucial for comparative genomics research, are not curated or verified for accuracy (16). This is further complicated by data omissions, poorly controlled sample description terminology, variable taxonomic naming conventions, and competing metadata package formats during submission that require different fields. Indeed, these points have all underpinned several recent studies investigating inconsistencies among “reference genomes” and type strains for a variety of bacterial species (17–21). In many cases, tracing the provenance of an individual assembly to its source material in order to verify its authenticity becomes challenging, and manual curation is frequently required to detect and correct these metadata errors (22). While it is not within the scope for RefSeq to present only the most up-to-date and accurate sequences available, it does place the burden of data accuracy on end users (as opposed to depositors) in recognizing and properly accounting for genome assembly and metadata accuracy issues.

Here, we present the results of an ongoing whole-genome sequencing (WGS) initiative at ATCC to provide end-to-end data provenance from source materials to reference-grade microbial genomes, here referred to as ATCC standard reference genomes (ASRGs). Although over 2,000 ASRGs are currently available from the ATCC Genome Portal, the 1,113 bacterial ASRGs presented here represent those that were available when this study was concluded. We compared these assemblies to those in RefSeq where metadata indicated they were produced by 3rd-party labs that sourced their materials from ATCC. For 366 ASRGs (~33%), we were able use metadata to compare them to one or more assemblies in RefSeq. The remaining 747 ASRGs (~66%) represent assemblies for bacterial strains that do not have clear counterparts for the same strains in RefSeq. All ASRGs described here are available for noncommercial research use via the ATCC Genome Portal (https://genomes.atcc.org) (23).

## RESULTS

### Whole-genome sequencing of 1,113 ATCC bacterial strains.

**FIG 1:**
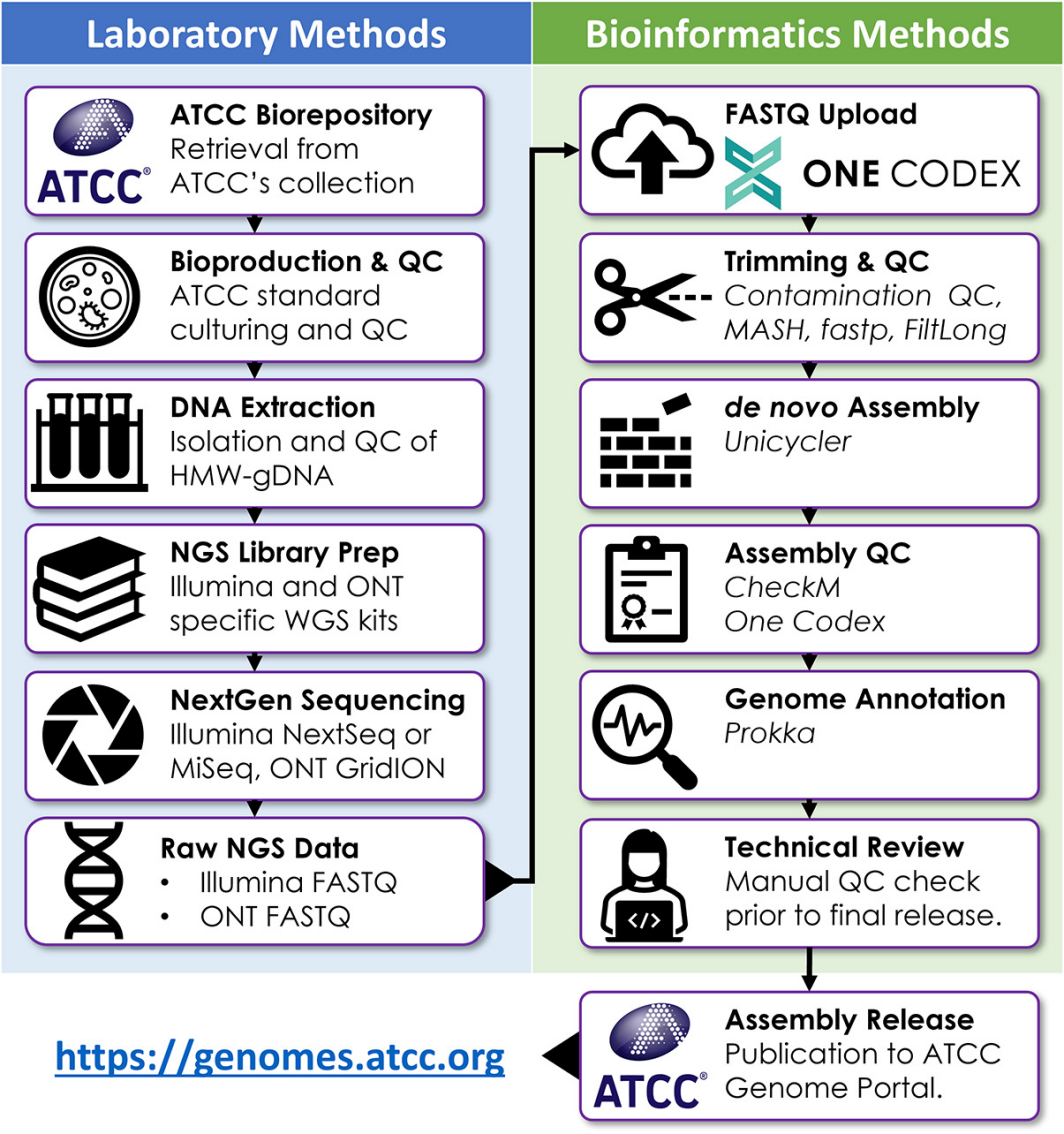
Pipeline for end-to-end genomic data provenance. Source materials were obtained directly from the ATCC biorepository and tracked through to the final assembly and genome annotation. Upfront culture conditions varied depending on the species cultured, but downstream process steps were performed using standardized protocols for DNA extraction, library prep, sequencing, and bioinformatics. Each pipeline is hosted on One Codex’s cloud infrastructure.

**FIG 2:**
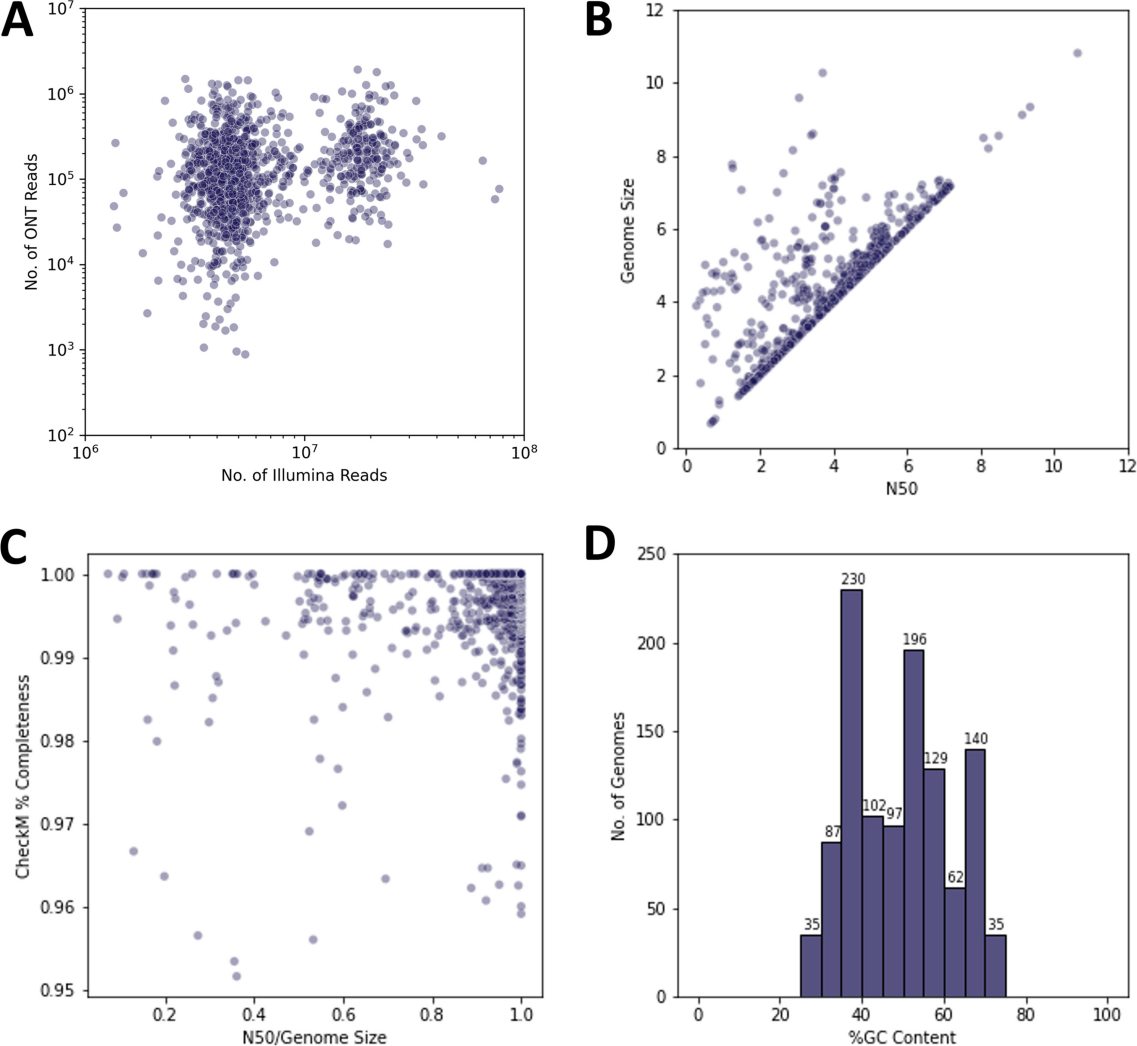
Sequencing and quality metrics for 1,113 bacterial genome assemblies. (A) Illumina versus ONT reads for ASRGs before down-sampling; (B) *N*_50_ metrics versus genome size; (C) *N*_50_ normalized by genome size versus *CheckM* genome completion estimates; (D) diversity of GC content for all 1,113 ASRG assemblies.

High-molecular-weight genomic DNA (HMW-gDNA) was extracted from 1,113 bacterial strains obtained from ATCC’s biorepository and subjected to hybrid whole-genome sequencing (WGS), assembly, and deposition to the ATCC Genome Portal. Briefly, strain selection was based on a combination of the number factors, including frequency of requests from ATCC’s repository over the last 5 years, inventory availability, biosafety level, and specific researcher requests. This resulted in 11 different bacterial phyla being included in this study. Briefly, each strain was cultured using strain-specific protocols and subjected to quality control (QC) for contamination, viability, purity, phenotype, and taxonomic identity (Fig. 1). For WGS, HMW-gDNA was split and subjected to sequencing using both Illumina and Oxford Nanopore Technologies (ONT) next-generation sequencing (NGS) platforms (Fig. 1). Next, reads were taxonomically classified using the One Codex platform to assess the purity of each NGS library prior to *de novo* assembly (24). Read sets were then down-sampled to predetermined coverage depths (Illumina, 100×; ONT, 60×) expected to be optimal for bacterial genome assemblies (25–28). Lastly, a hybrid assembly pipeline incorporating reads from both platforms produced *de novo* assemblies for each strain using *Unicycler* (28). High-level summary metrics for each ASRG are shown in Fig. 2 and Table S1 in the supplemental material. All 1,113 ASRG assemblies were estimated to be over 95% complete by *CheckM*; 1,015 were found to be over 99% complete and 329 are 100% complete (29). A total of 617 are considered high-quality, closed genome references.

10.1128/msphere.00077-22.5 TABLE S1 High-level summary metrics for each ATCC standard reference genome (ASRG), including the following columns: NCBI taxonomy name, ATCC strain name, indicator for type strain, GC content, filtered *N*_50_ value, genome assembly size, ratio of *N*_50_ to genome assembly size, total number of contigs per assembly, *CheckM* percent estimate of genome completeness, total number of Illumina reads, total number of Nanopore reads, and *CheckM* contamination percentage estimate. Download Table S1, XLSX file, 0.1 MB. Copyright © 2022 Yarmosh et al. 2022 Yarmosh et al. https://creativecommons.org/licenses/by/4.0/ This content is distributed under the terms of the Creative Commons Attribution 4.0 International license.

### Survey of bacterial genome assemblies in RefSeq.

**FIG 3:**
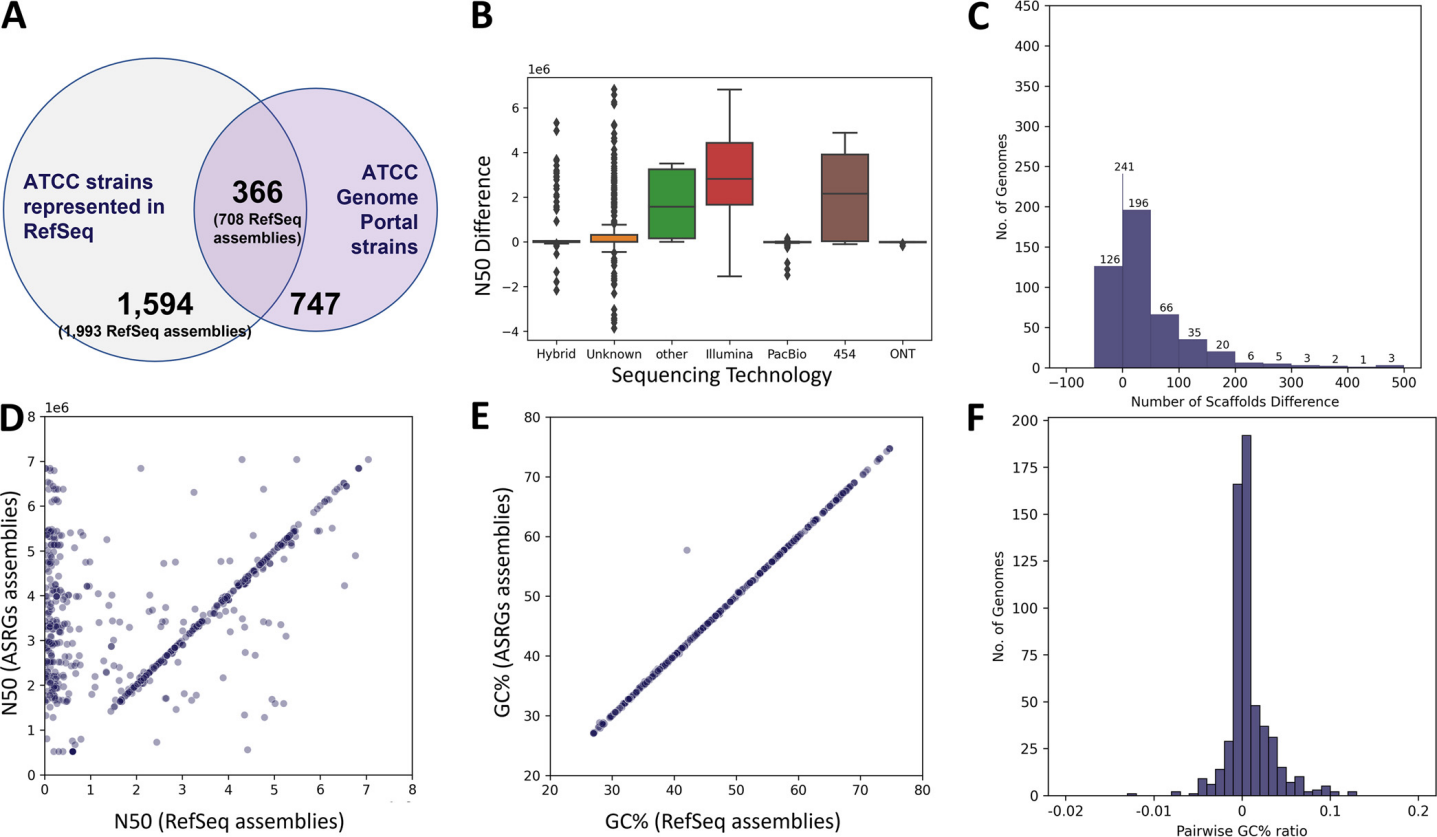
Comparative metrics for 1,113 ASRGs versus RefSeq Assemblies. (A) Intersection of ASRGs versus RefSeq for strains labeled as being from ATCC. In parentheses are the total numbers of RefSeq assemblies, allowing for strain redundancy. (B) *N*_50_ variability of RefSeq versus ASRGs by sequencing technology. Note that the scale is 1E6. (C) Differences in contig counts for ASRG versus RefSeq assemblies. Positive values indicate that the RefSeq assembly had more contigs. (D) Ratios of ASRG *N*_50_ values (*y* axis) to RefSeq *N*_50_ values (“public,” *x* axis). Density along the diagonal indicates that many assemblies are similar, while density along the *y* axis indicates ASRGs with higher *N*_50_ values. (E) GC content for ASRGs (*y* axis) versus RefSeq (*x* axis). Nearly all assemblies have less than 0.1% difference in GC content. (F) Pairwise GC content differences between ASRGs and comparable RefSeq assemblies for the same strain.

We compared the ASRG assemblies to those in NCBI’s RefSeq bacterial database (https://ftp.ncbi.nlm.nih.gov/genomes/refseq/bacteria/) labeled as representing ATCC bacterial strains, i.e., assemblies where the ATCC strain name (or a synonymous name) was indicated in the title, description, or organism name fields in the GenBank assembly record. We intentionally did not search RefSeq using a traditional comparative genomics approach (i.e., by sequence homology, BLAST, etc.) since this would require arbitrary thresholds for determining strain identity, and metadata descriptors are intended to be useful for these types of queries. Using this approach, we found 2,701 genome assemblies in RefSeq, which collectively comprised 1,960 different ATCC strains (Fig. 3A and Table S2). Interestingly, RefSeq had numerous examples of bacterial strains represented by multiple assemblies or submitted by different groups, and it often included “type strains” resulting from intentional genetic modification (e.g., there are 33 different RefSeq assemblies for Serratia marcescens subsp. *marcescens* ATCC 13880). This is despite it representing a “nonredundant” database (although the specific meaning of this is not clearly defined) (9). Moreover, while each of these 33 assemblies have fields in the assembly record describing them as genetically modified genomes, each is also labeled as “assembled from type material.” Overall, we found one or more duplicate assemblies in RefSeq for 158 strains for which we also produced an ASRG, including instances of assemblies for genetically modified strains mislabeled as representing type strains (see Table S2). These errors and strain duplications create risks for researchers who may unwittingly use these data in their own research yet remain unaware of these issues.

10.1128/msphere.00077-22.6 TABLE S2 High-level summary of preexisting NCBI genome assemblies for ATCC bacterial strains included in this study. Table includes NCBI taxonomy name, RefSeq assembly accession identifier, indicator for alternative assembly on the ATCC Genome Portal, ATCC strain name, assembly genome coverage, assembly release type, RefSeq assembly category, submitter identifier, BioProject identifier, WGS project identifier, GenBank assembly accession identifier, reference guided assembly details, isolate details, assembly type details, organism name, infraspecific name, assembly method, genome representation, NCBI taxonomy identifier, submitter assembly name, relation of assembly to type material, BioSample identifier, sequencing technology description, date assembly submitted, and assembly level. Download Table S2, XLSX file, 0.5 MB. Copyright © 2022 Yarmosh et al. 2022 Yarmosh et al. https://creativecommons.org/licenses/by/4.0/ This content is distributed under the terms of the Creative Commons Attribution 4.0 International license.

Further examination of the metadata for the 2,701 RefSeq assemblies labeled as ATCC strains also revealed numerous records with incomplete, missing, or obscured descriptor fields (Fig. S1). For example, “assembly type” is present in every assembly record but the entry is “na” for all. “sequencing technology” is not included or has an entry of “unknown” for 1,088 assemblies (~40% [Table S2]), and spelling and nonstandard abbreviations further complicate the rest. **“**Assembly method” is not included for 1,082 assemblies, contains the entry “unknown**”** for 88 assemblies or “other” for four assemblies, and has numerous misspellings for various bioinformatics tools (i.e., “Velevt” or “Velveth” for the Velvet assembler). One example (GCF_015708605.1) simply indicates the “assembly method” as “several assembly pipelines, manual curation v. 2018-09-27.” Underutilized fields included “description,” “isolate,” and “relation to type material,” which had no entries in 99%, 98%, and 38% of the assembly records, respectively. The damaging impact that inconsistent depositor metadata has on scientific research and reproducibility has been extensively covered elsewhere (1, 3, 30). Within the context of this study, these metadata gaps reveal difficulties in adequately identifying the appropriate RefSeq sequences for further research.

10.1128/msphere.00077-22.1 FIG S1 Bar chart demonstrating the percentage of RefSeq assembly report fields that are left empty or contain “na” as an entry. While some of these, such as RefSeq category, have implicit definitions for empty fields, others, such as relation to type material, are potentially crucial pieces of information. Download FIG S1, TIF file, 1.4 MB. Copyright © 2022 Yarmosh et al. 2022 Yarmosh et al. https://creativecommons.org/licenses/by/4.0/ This content is distributed under the terms of the Creative Commons Attribution 4.0 International license.

Of the 2,701 RefSeq assemblies for ATCC bacterial strains, 708 had a counterpart ASRG (Fig. 3A and Table S2). Of these, 303 (43%) are labeled “complete genome” or “chromosome” level assemblies. Despite this, *N*_50_ values were largely inferior to their ASRG counterparts (Fig. 3B). While 241 RefSeq assemblies had the same number of scaffolds as their corresponding ASRGs, 341 were more fragmented. Altogether, 662 ASRGs had *N*_50_ values equivalent or superior to their RefSeq counterparts (ATCC *N*_50_/RefSeq *N*_50_ ≥ 0.95), while 46 ASRG assemblies were more fragmented (Fig. 3D). The greatest difference was observed for a RefSeq assembly for Pseudomonas aeruginosa ATCC 700888 (GCF_000297315.1), which comprised 600 contigs, while the ASRG equivalent is a closed and finished genome, containing a single replicon. Because this Pseudomonas strain was submitted to RefSeq in 2012 after having been sequenced on an Illumina GAIIx, it is no surprise that our assembly (produced with both Illumina and Nanopore data) produced superior assembly. Nonetheless, arbitrarily excluding this genome from our comparisons also would not befit the study, as one of our primary goals was to investigate the overall quality of assemblies and their corresponding metadata independent of the context, timing, or relative perceived importance of each of them.

### Comparative genomics of 303 RefSeq assemblies.

Next, we compared the 303 complete RefSeq assemblies to their corresponding ASRGs for the same strains (represented by 212 ASRGs). First, we found that the pairwise average nucleotide identity (ANI) ranged from 97% to 100% for identical strains, which at first glance suggested a high level of similarity (31). Although large differences in the high-level assembly metrics were previously observed (e.g., *N*_50_ and GC content), after conducting pairwise whole-genome alignments with *MUMmer4* for all 303 RefSeq assemblies against ASRGs for the same strain, we found that 292 had over 95% of their sequence aligned. Next, we examined pairwise structural variations and found significant differences in sequence repeats, inversions, insertions/deletions (indels), and translocations between RefSeq assemblies and ASRGs for the same strains (Tables S3 and S4) (32). Analysis with *dnadiff* of all 303 RefSeq assemblies revealed an average 6.73 structural rearrangements in comparison to ASRGs, the worst of which was GCF_000160895.1 for Bacillus cereus ATCC 10876, with 232 structural differences (despite both assemblies having over 99% reciprocally aligned bases). Structural relocations were the most common, with 256 RefSeq assemblies having at least 1 per assembly (average, 4.3 per assembly). Structural inversions were found in 74 RefSeq assemblies (average, 2.2). Translocations were relatively rare, with only 9 RefSeq assemblies having structural translocations relative to the ASRG assembly for the same strain (Table S4). We also found that RefSeq assemblies with the greatest number of structural differences from the ATCC assemblies corresponded to those submitted to NCBI prior to 2010 and for which “sequencing technology” or “assembly method” was not indicated in the RefSeq metadata. The distribution of structural variations in the 303 complete RefSeq assemblies compared to their corresponding ASRGs is shown in Fig. S2.

10.1128/msphere.00077-22.2 FIG S2 (A) Stacked bar chart showing relocation, inversion, and translocation structural variants between all ATCC assemblies and assemblies generated from mapping ATCC’s read data of specific strains to assemblies of those strains; (B) stacked bar chart showing relocation, inversion, and translocation structural variants between ATCC assemblies and assemblies generated from mapping ATCC’s read data of specific strains to assemblies of those strains for the 50 ATCC products with the greatest total of structural variants. Download FIG S2, TIF file, 0.9 MB. Copyright © 2022 Yarmosh et al. 2022 Yarmosh et al. https://creativecommons.org/licenses/by/4.0/ This content is distributed under the terms of the Creative Commons Attribution 4.0 International license.

10.1128/msphere.00077-22.7 TABLE S3 Joint table of comparing assemblies following serial pairwise reciprocal whole-genome alignments with *MUMmer4* (see Materials and Methods) of RefSeq assemblies (column B) to ATCC standard reference genome assemblies (column C). Table includes NCBI taxonomy name, ATCC catalog number, RefSeq assembly level, RefSeq assembly scaffold count, ATCC assembly scaffold count, log ratio of ATCC and RefSeq scaffold counts, *N*_50_ of RefSeq assembly, *N*_50_ of ATCC assembly; log ratio of ATCC and RefSeq *N*_50_ values, total length of RefSeq assembly, total length of ATCC assembly, log ratio of ATCC and RefSeq assembly lengths, GC_RefSeq, GC_ATCC, and log ratio of GC values. Download Table S3, XLSX file, 0.1 MB. Copyright © 2022 Yarmosh et al. 2022 Yarmosh et al. https://creativecommons.org/licenses/by/4.0/ This content is distributed under the terms of the Creative Commons Attribution 4.0 International license.

10.1128/msphere.00077-22.8 TABLE S4 Joint table of comparing assemblies following serial pairwise reciprocal whole-genome alignments with *MUMmer4* (see Materials and Methods) of RefSeq assemblies (column B) to ATCC standard reference genome assemblies (column C). Table includes NCBI taxonomy name, ATCC catalog number, RefSeq reference ID, RefTotalBases aligned, QueryTotalBases aligned, RefSequences aligned, QuerySequences, RefAlignedBase%, QueryAlignedBase%, RefRelocations, QueryRelocations, RefTranslocations, QueryTranslocations, RefInversions, QueryInversions, RefTotal SV, and average nucleotide identity (ANI). Column names are from *MUMmer4* output, but “Ref…” and “Query…” in these columns refer to RefSeq and ATCC genome assemblies, respectively. Download Table S4, XLSX file, 0.05 MB. Copyright © 2022 Yarmosh et al. 2022 Yarmosh et al. https://creativecommons.org/licenses/by/4.0/ This content is distributed under the terms of the Creative Commons Attribution 4.0 International license.

### Variants in 303 RefSeq assemblies.

Next, we sought to investigate the prevalence of single-nucleotide polymorphisms (SNPs) and indels that would arise by using RefSeq assemblies as a reference genome against which Illumina sequencing data would be mapped—a common approach used by labs without the resources or expertise for *de novo* assembly and annotation. For each of the 303 complete RefSeq assemblies described above, we mapped the same Illumina reads used in creating the corresponding ASRGs for the same strain. Variant calling from the resulting consensus genomes was carried out on all 303 references to detect SNPs and indels in each (see Materials and Methods). Overall, the number of SNPs and indels per assembly ranged from 0 (none detected) to as many as 60,064 SNPs (Acinetobacter baumannii ATCC 17978, GCF_011067065.1) and 2,699 indels for a given assembly (Parabacteroides distasonis ATCC 8503, GCF_900683725.1) (Table S5). The median level of SNPs and indels was 7 SNPs and 8 indels per assembly, with 7 of the 303 mappings having no detectable SNPs and indels. These results were promising overall, yet significant outliers were detected, and 26 strains had SNPs and indels beyond an extreme-outlier boundary, i.e., greater than 3 times the interquartile range (IQR) above the median, with 9 of them having over 1,000 SNPs and indels each (Fig. S4a and b and Fig. S5).

10.1128/msphere.00077-22.4 FIG S4 A visualization of the number and types of variants found when mapping the trimmed Illumina reads for an ATCC product to its corresponding RefSeq assembly/assemblies. (A) Total number of variants, number of SNPs, and number of indels found across this mapping; (B) total number of variants and characterization of those variants as either synonymous or nonsynonymous, as determined by the Ensembl Variant Effect Predictor (VEP). Synonymous variants represent alterations to a coding sequence that does not change the amino acid upon translation. Nonsynonymous variants represent alterations to a coding sequence that does change the amino acid upon translation. Variants outside coding regions were calculated as well but are not shown. Download FIG S4, TIF file, 1.8 MB. Copyright © 2022 Yarmosh et al. 2022 Yarmosh et al. https://creativecommons.org/licenses/by/4.0/ This content is distributed under the terms of the Creative Commons Attribution 4.0 International license.

10.1128/msphere.00077-22.9 TABLE S5 Joint table of read mapping and variant calling results of ATCC Illumina sequencing data mapped against available RefSeq assembly genomes (see Materials and Methods). Results include NCBI taxonomy name, ATCC catalog number, RefSeq reference identifier for mapping, RefSeq assembly level, RefSeq category identifier, Illumina coverage for ATCC sequencing reads, number of SNPs called, number of indels called, total number of unique variants, total synonymous variants, total nonsynonymous variants (including missense variants, frameshift variants, and variants from missing start or stop codons), total other variants (including no annotations, intergenic variants, and upstream and downstream gene variants within 1,000 nucleotides), and total number of identified duplicate variants. Download Table S5, XLSX file, 0.04 MB. Copyright © 2022 Yarmosh et al. 2022 Yarmosh et al. https://creativecommons.org/licenses/by/4.0/ This content is distributed under the terms of the Creative Commons Attribution 4.0 International license.

A total of 111 assemblies had fewer than 10 variants (SNPs and indels), while 15 assemblies had more than 500 variants (SNPs and indels). Not surprisingly, as the number of SNPs increased, so too did the number of indels (Fig. S3). Of these, 52 of the 303 assemblies had no expected nonsynonymous mutations, but 87 had at least 10 nonsynonymous variants per genome (Fig. S4b). Importantly, 52 RefSeq assemblies identified as “assembled from type material” were found to have at least 10 nonsynonymous variants, and 7 assemblies had over 100; this could have potentially deleterious impacts on future comparative genomics studies utilizing those reference assemblies (Table S5).

10.1128/msphere.00077-22.3 FIG S3 Single-nucleotide polymorphisms (SNPs) and insertions/deletions (indels) of ASRG raw data mapped to RefSeq references. Each data point represents a read mapping of ASRG raw data (Illumina only) to a RefSeq genome assembly for the same bacterial strain. In cases where multiple RefSeq assemblies exist for the same bacterial strain, ASRG reads were mapped to each and are represented above by multiple data points. The extreme outlier boundary (red) is determined as 3 times the interquartile range above the median for both SNPs and indels (see Materials and Methods in the main text). Download FIG S3, TIF file, 1.6 MB. Copyright © 2022 Yarmosh et al. 2022 Yarmosh et al. https://creativecommons.org/licenses/by/4.0/ This content is distributed under the terms of the Creative Commons Attribution 4.0 International license.

We found that RefSeq assemblies without the label “reference genome” or “representative genome” (250 genomes) were enriched for SNPs (7.6-fold) and indels (9.6-fold) compared to complete reference RefSeq genomes (53 assemblies). Furthermore, type strain assemblies in RefSeq (i.e., labeled as “assembly designated as neotype,” “assembly from synonym type material,” or “assembly from type material”) had generally had fewer SNPs and indels than other assemblies overall, but some significant exceptions to this were also observed (see above). No statistically significant enrichment for SNPs or indels was detectable by taxonomic clade or GC content. Collectively, these results underscore the importance of either manual curation (e.g., “reference genome” or “representative genome”) or data provenance of the originating materials (e.g., “assembled from type strain material”) and that they are both important drivers in reducing variability and improving published genome assembly quality.

## DISCUSSION

Over the last 20 years, several noncommercial and government initiatives have specifically tried to address issues relating to the quality and standardization of metadata for microbial genomics, which has had some benefit for end users, but substantial work remains to be done (15, 33, 34). As the unmet need for curated, high-quality microbial genomics data continues to grow, we will no doubt continue to see commercial initiatives designed to address gaps in quality, content, and reliability, such as Qiagen’s CLC Microbial Reference Database, ARES Genetics’ ARESdb, and the One Codex platform (24, 35, 36). While these public and private efforts have been relatively successful, others have raised concerns about declines in public microbial genomics metadata (2, 3, 5, 11, 14, 37). We assert that widespread gaps in the traceability of genome assemblies to their originating biological materials, lab protocols, and bioinformatics methods represent a fundamental weakness in these data that hinders research reproducibility and progress. Further, databases such as RefSeq, that do not aim for completely attributable data, are potentially being misused in research studies where provenance and authenticity are assumed. We are attempting to address these gaps for ATCC strains by reestablishing the provenance of these data to physical materials held within ATCC’s biorepository and making these data available for research use purposes via the ATCC Genome Portal (23).

At the outset of the work described here, we sought to systematically sequence ATCC’s bacterial collection—which has become an ongoing initiative that has recently expanded to also include viruses and fungi. However, during the course of our work, we found that bacterial genome assemblies in RefSeq labeled as representing ATCC strains poorly compared against the ASRGs we produced in-house, both in terms of genome assembly metrics and as they related to strain and assembly metadata. Although sequencing technologies have improved dramatically over the last 2 decades, what surprised us in our initial analysis were the disparities in the quality, accuracy, traceability, and completeness of metadata associated with RefSeq assemblies—which are largely technology independent. It is out of the scope of this study to suggest mechanisms by which NCBI could further control for these concerns, but it does highlight the need for users to consider databases that seek to accurately and consistently capture this information. We found that gaps in data provenance are playing a role in the poor data quality overall. The number of incomplete records increases over time and records are not regularly replaced with more complete versions, which we feel underscores the importance of the genomics initiatives at ATCC. As an example, over 33% (1,087) of the RefSeq assemblies included in our study completely lacked any description for how they were sequenced or assembled in the first place. Furthermore, among the 584 institutions listed among the BioProjects containing assemblies for ATCC bacterial strains (Table S2), only 85 (14.5%) of those institutions definitively obtained these strains directly from ATCC, which no doubt has a negative impact on the traceability and quality of genome assemblies found within RefSeq. Although it is an estimate, due to institutional name changes or potentially other issues, this nonetheless highlights gaps in our understanding of the source of the strains used to produce these assemblies and the historical provenance of the data associated with them, despite being labeled as a representative or reference genomes for ATCC strains.

There are myriad reasons for why the ASRGs presented in this study generally outperform their counterparts in RefSeq. Obviously, next-generation sequencing technology has improved significantly over the last decade on all points. In addition, *de novo* assembly software is continuously improving, and ATCC developed a standardized workflow for all ASRGs—which is typically not done by research-focused groups. Further, ASRGs are produced directly from biomaterials traceable to physical inventory lots held within ATCC’s biorepository in an ISO 9000- and ISO 17025-compliant laboratory, which collectively serves to reduce the opportunity for lab-acquired adaptations, strain domestication, incorrect sample labeling, sample or library contamination, breaks in provenance, and other disruptions in authenticity. It is not within RefSeq’s mission to account for these sources of error, nor is it within the mission of most publicly accessible genome databases, but it does represent a gap that is often overlooked by many and that may contribute significantly to issues related to scientific reproducibility. In a broader context, the microbial research community would benefit from establishing a standard for documenting the provenance of physical cultures, isolates obtained from them, and data derived from those isolates (e.g., genomics data). Prior efforts by other groups, such as AOAC International’s Stakeholder Panel on Agent Detection Assays (SPADA) Working Group for Bacterial Strain Verification, have attempted to establish provenance standards for physical strains and isolates. In contrast, no formal standard has been proposed for genomics data provenance and authenticity. Specifically, the “source material” attribute of the MIGS genomics metadata standard is defined as being “optional,” which perhaps represents a missed opportunity to improve the quality and traceability of genomics research data (15, 38).

In general, researchers should be cautious about the data they obtain from public databases and avoid blindly ingesting reference genome data without first being curious about the origins of the data, the methods used to produce them, and the completeness of the associated metadata. We suggest that for assemblies that are named after and represent culture collection strains or type strains, the highest level of assembly quality, metadata completeness, and data provenance should be expected from depositors; otherwise, many of the issues we have described above will continue to persist. Simply assuming that domestication or laboratory adaptation of strains accounts for the variability observed in RefSeq and GenBank assemblies is insufficient, as these differences can often result in real-world phenotypic changes not reflected in the strains held within culture collections themselves (17–19, 21). We want to encourage data depositors and researchers to continue to use culture collection identifiers whenever submitting new assemblies for strains held within these collections, but we also urge the administrators of public genome databases to place a higher level of accountability on the completeness and quality of these submissions. Doing so would serve to improve the overall reliability of these public resources and reduce the amount of postsubmission curation done by end users wishing to use these data directly in their research. Lastly, we suggest that further systematic studies are needed to better understand the risks and prevalence of inauthentic and inaccurate data found in microbial genomics databases, and the impact they have on basic and applied research. It is our hope that initiatives focused on genomic data provenance, such as the work presented above, will serve to highlight the value of establishing higher standards for traceability and accountability in public microbial genomics databases.

## MATERIALS AND METHODS

### Sample acquisition and culture conditions.

All the bacterial cell cultures and genomic DNA used in this study met or exceeded ATCC’s quality standards (https://www.atcc.org/about-us/quality-commitment), underwent extensive phenotypic and genotypic characterization to ensure accurate strain identification, and were extensively tested for contamination before being accepted for use in this study. ATCC is certified by the ANSI National Accreditation Board (ANAB) to meet both ISO 17034:2016 standards as a reference material producer and ISO/IEC 17025:2017 as a testing and calibration reference laboratory. Each bacterial strain included in this study is available from ATCC’s biorepository and was authenticated according to protocols executed in accordance with ATCC’s quality management system (see above). The specific protocols for each strain varied depending on the specific species in question. In general, strain identification and authentication included assessment of colony morphology, Gram staining, culture purity, metabolic profiling, antibiotic susceptibility testing (AST), broad-spectrum biochemical reactivity testing, 16S rRNA gene sequencing, ribotyping, matrix-assisted laser desorption ionization–time of flight mass spectrometry (e.g., bioMérieux Vitek MS system), and whole-genome next-generation sequencing (NGS). Additional details used for culturing, growth conditions, and authentication of each bacterial strain are available online in each bacterial strain’s catalog page at https://www.atcc.org/ and by visiting ATCC’s Bacterial Cell Culture portal (39).

### DNA templates and quality control.

To facilitate the successful NGS library preparation for multiple sequencing platforms (long- and short-read sequences), both high-quality and high-quantity input DNA was obtained from authenticated genomic DNA (gDNA) available in ATCC bacterial nucleic acids repository (40). ATCC uses several commercially available extraction kits and in-house-validated protocols to obtain pure high-molecular-weight DNA depending on the biological characteristics of the organism undergoing extraction. For strains with no preexisting genomic DNA in ATCC’s repository, total high molecular weight genomic DNA (HMW-gDNA) was extracted from thawed or resuspended frozen cultures with 10^7^ to 10^9^ cells/mL using the Qiagen Genomic-Tip 20/g or 100/g kit and analyzed for purity, concentration, and fragment size. HMW-gDNA samples meeting or exceeding the following criteria were subjected to sequencing: median fragment size larger than 20 kb, optical density *A*_260_/*A*_280_ between 1.75 and 2.00, and a final elution concentration over 20 ng/μL per extraction.

### Short-read next-generation sequencing.

High-quality gDNA from each strain was subjected to whole-genome sequencing using a short-read NGS workflow. Briefly, sequencing libraries from each extraction were prepared using the DNA prep kit, indexed using DNA/RNA UD indexes (Illumina), and subsequently subjected to paired-end sequencing on either an Illumina MiSeq or NextSeq 2000 instrument. Sample multiplexing was based on achieving a minimum 100× average depth of coverage for each genome. Base-calling and adapter trimming were initially done using onboard Illumina instrument software and followed by an additional round of trimming and quality score filtering using *fastp* with default settings and *FastQC* (26, 41). Illumina reads accepted for further use passed the following quality control thresholds: median Q score, >30 for all bases; median Q score, >25 per base; and ambiguous content (N bases), <5%. The versions of bioinformatics software used throughput this study are listed in Table S6.

10.1128/msphere.00077-22.10 TABLE S6 Summary table of bioinformatics pipelines, software versions, and their parameters. Further details are available on our GitHub site (see “Data availability” in the main text). Download Table S6, XLSX file, 0.02 MB. Copyright © 2022 Yarmosh et al. 2022 Yarmosh et al. https://creativecommons.org/licenses/by/4.0/ This content is distributed under the terms of the Creative Commons Attribution 4.0 International license.

### Long-read next-generation sequencing.

Long-read sequencing was carried out using the Oxford Nanopore Technologies (ONT) GridION platform. ONT ligation sequencing kit (SQK-LSK109) sequencing libraries were prepared from the same physical samples of HMW-gDNA as used for Illumina sequencing described above, multiplexed using the ONT native barcoding expansion kit (EXP-NBD104 or EXP-NBD114), and sequenced using GridION flow cells (ONT; R9.4.1). As with Illumina sequencing, the number of samples multiplexing was based on the estimated genome size of a given organism and sequencing was performed for a minimum of 48 h per flow cell. Using the most up-to-date version of *MinKNOW*, reads were base-called, using the high-accuracy settings, demultiplexed, and barcode trimmed. Furthermore, ONT sequencing reads were quality trimmed and filtered using *Filtlong* to meet the following minimum acceptance criteria: minimum mean Q score per read of >10 and minimum read length of >5,000 bp (42). *Filtlong* was run with the following settings: min_length 1,000, target_bases = genome size × Oxford Nanopore sequencing targeted depth.

### Assembly of ATCC standard reference genomes.

For genome references deposited to the ATCC Genome Portal, genome assembly size was first estimated from raw reads using *MASH*, and this estimate was used to down-sample the Illumina and ONT raw sequencing libraries to maximum 100× and 40× coverages, respectively (43). These coverage requirements were selected to maximize accuracy for individual consensus base calls in the final assemblies (25, 27). After down-sampling each sequencing library, a hybrid *de novo* assembly approach was taken using *Unicycler* with default settings (28). Briefly, Illumina libraries were first assembled individually into contigs. The longest contigs in the initial set were then scaffolded with reads from the ONT library. The combined hybrid assembly was then iteratively polished using both long and short reads from both input libraries, resulting in highly contiguous or closed reference genomes. Sequencing and assembly artifacts of less than 1,000 bp that also had significantly different coverage depth (e.g., “chaff” contigs) were removed from the final draft reference (44). These draft assemblies were subsequently checked using One Codex to confirm the species (24). Finally, each draft assembly was assessed for completeness and potential contamination with *CheckM lineage_wf*, which is based on orthologous gene copy numbers present in an assembly (29). Assemblies which were determined to have a *CheckM* “completeness” score above 95% and a contamination value below 5% were deemed final assemblies. Each final assembly was subsequently annotated using *Prokka* with default settings for coding DNA sequence (CDS), rRNA, tRNA, signal leader peptide, and noncoding RNA identification (45). Parameters for the various tools are shown in Table S6. Finally, each complete and annotated genome was deposited into the ATCC Genome Portal and is referred to here as an ATCC standard reference genome (ASRG) (23).

### Characterization of public genome assemblies.

To gather the public assemblies of ATCC bacterial strains, the “assembly_summary_refseq.txt” file was downloaded from NCBI via FTP (https://ftp.ncbi.nlm.nih.gov/genomes/refseq/bacteria/), accessed July 2019. This file contains accession numbers and metadata, such as “isolate,” “assembly level,” and “tax ID,” for every assembly in NCBI bacterial RefSeq. First, this file was filtered to keep all records that contained either the “ATCC” or “NCTC” keyword. This was done because many strains have synonymous ATCC and NCTC identifiers (IDs), though often only one of the two is present in a record. Of the records containing “ATCC” or “NCTC,” all that included “ATCC” were kept, but records containing “NCTC” were filtered to keep only those with a synonymous ATCC ID. This final set of records contained the 2,701 public assemblies of ATCC strains. While “assembly_summary_refseq.txt” does contain metadata, the complete set of metadata was collected by downloading the “assembly_report.txt” for each assembly from the NCBI ftp site. Metadata comparisons were performed using the *compare.all.levels.py* script after appending the RefSeq assembly data with a GC content column, calculated by *bbnorm_stats.sh*, all of which was paralleled with GNU Parallel (46). ATCC’s Genome Portal does not distinguish between contigs and scaffolds, which RefSeq defines as contigs that are connected across gaps. For this, all data comparison of ASRGs in terms of contiguity uses RefSeq scaffold information.

### Comparisons of NCBI and ATCC genome assembly metrics.

For each of the bacterial strains included in the ATCC Genome Portal, we identified and downloaded all 2,701 genome assemblies that had the same name or similar names from NCBI’s RefSeq and Genome Assembly databases. For the 303 NCBI assemblies with a finished assembly status of “complete” or “chromosome” and representation in ATCC’s Genome Portal, we carried out pairwise whole-genome alignments for each NCBI and ASRG using *MUMmer4* and its associated suite of tools for comparative genomics (32). In some cases, due to duplications in RefSeq and NCBI’s Genome Assembly database, multiple NCBI assemblies were compared against the same ASRG assembly. Following the creation of the alignments, we identified genome-wide variants for each NCBI assembly compared to the ASRG assembly, including single-nucleotide polymorphisms (SNPs), insertions and deletions (indels), and structural variants (SVs). Genome-wide comparisons using *dnadiff* included assembly length, number of contigs, pairwise percent aligned, and *N*_50_ values (SVs_and_ANI.sh) (47). Furthermore, *MUMmer4*’s *dnadiff* tool was run with default settings using the ASRG assemblies against each NCBI RefSeq assembly, and relocations, translocations, and inversions are reported alongside total and aligned bases (32). Prior to running *MUMmer4*’s *dnadiff* tool on these assemblies, each was filtered to remove contigs of <1kb in length to prevent short sequences from exaggerating SVs between assemblies. Structural variants included breakpoints, relocations, translocations, and inversions, and summarized as rearrangements.

### Read-Mapping and Variant Calling with NCBI Assemblies.

For reference based variant calling, 303 “complete genome” assemblies were downloaded from NCBI’s RefSeq that had corresponding ASRG assemblies and used as references for read-mapping and variant calling. Only the corresponding Illumina sequencing reads from each ASRG assembly (see above) was used as input. For each RefSeq genome, we mapped reads from a corresponding ASRG assembly using BWA-mem v0.7.17 with the default parameters (48). Quality metrics were recorded using Qualimap bamqc v2.2.1 (49). Variants were called using GATK Haplotype caller (v.4.1.8.1, standard minimum confidence threshold = 30) (50). The Ensembl Variant Effect Predictor (VEP) was used to identify synonymous and non-synonymous variants in each reference mapping (51). The complete pipeline is available on our GitHub repository.

### Data availability.

Source code for ATCC scripts is available at https://github.com/ATCC-Bioinformatics/Equivalency_Analysis. Raw NGS data (FASTQ files) are available for research use only from https://github.com/ATCC-Bioinformatics/AGP-Raw-Data/blob/main/AGP_Raw-Data-Access.txt. ASRGs and associated metadata are available directly from the ATCC Genome Portal (https://genomes.atcc.org) or via our REST-API (access details available upon request).
