# Supplementary figures and images for: Comparative Analysis and Data Provenance for 1,113 Bacterial Genome Assemblies
Source: mSphere. 2022 May 2;7(3):e00077-22. doi: 10.1128/msphere.00077-22 (PMC9241530; doi:10.1128/msphere.00077-22)

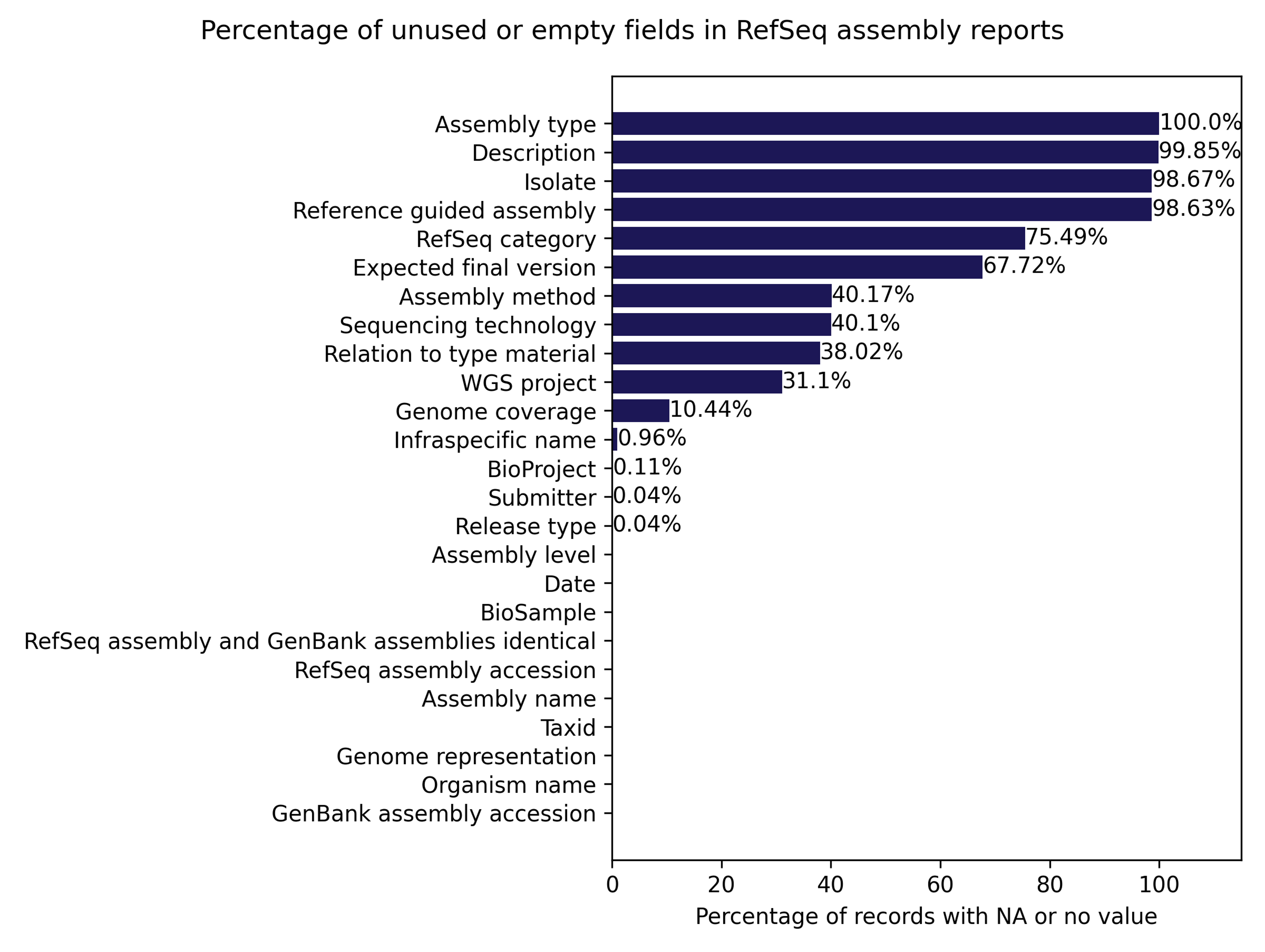

Supplement: FIG S1 [file msphere.00077-22-s0001.tif]

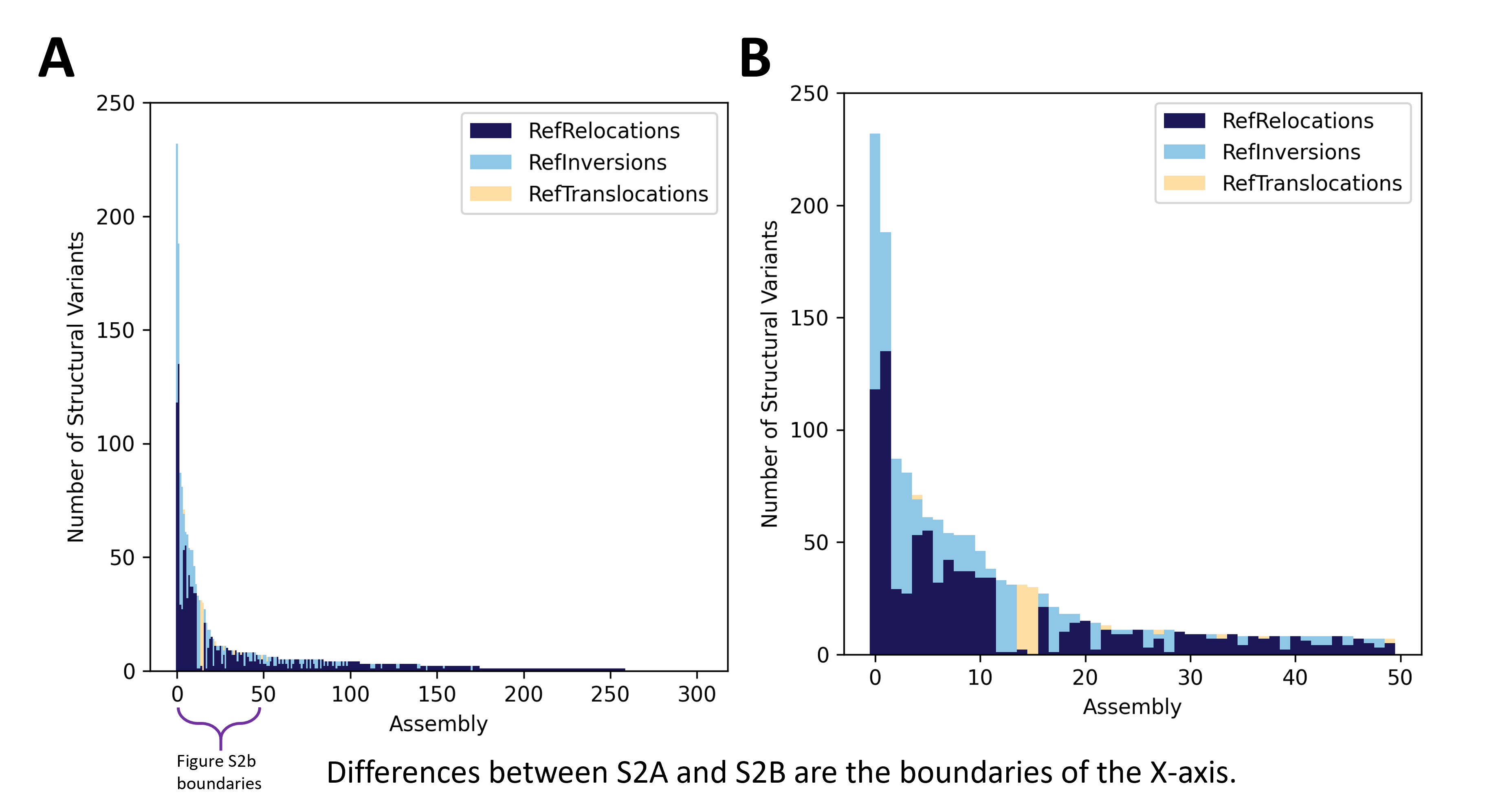

Supplement: FIG S2 [file msphere.00077-22-s0002.tif]

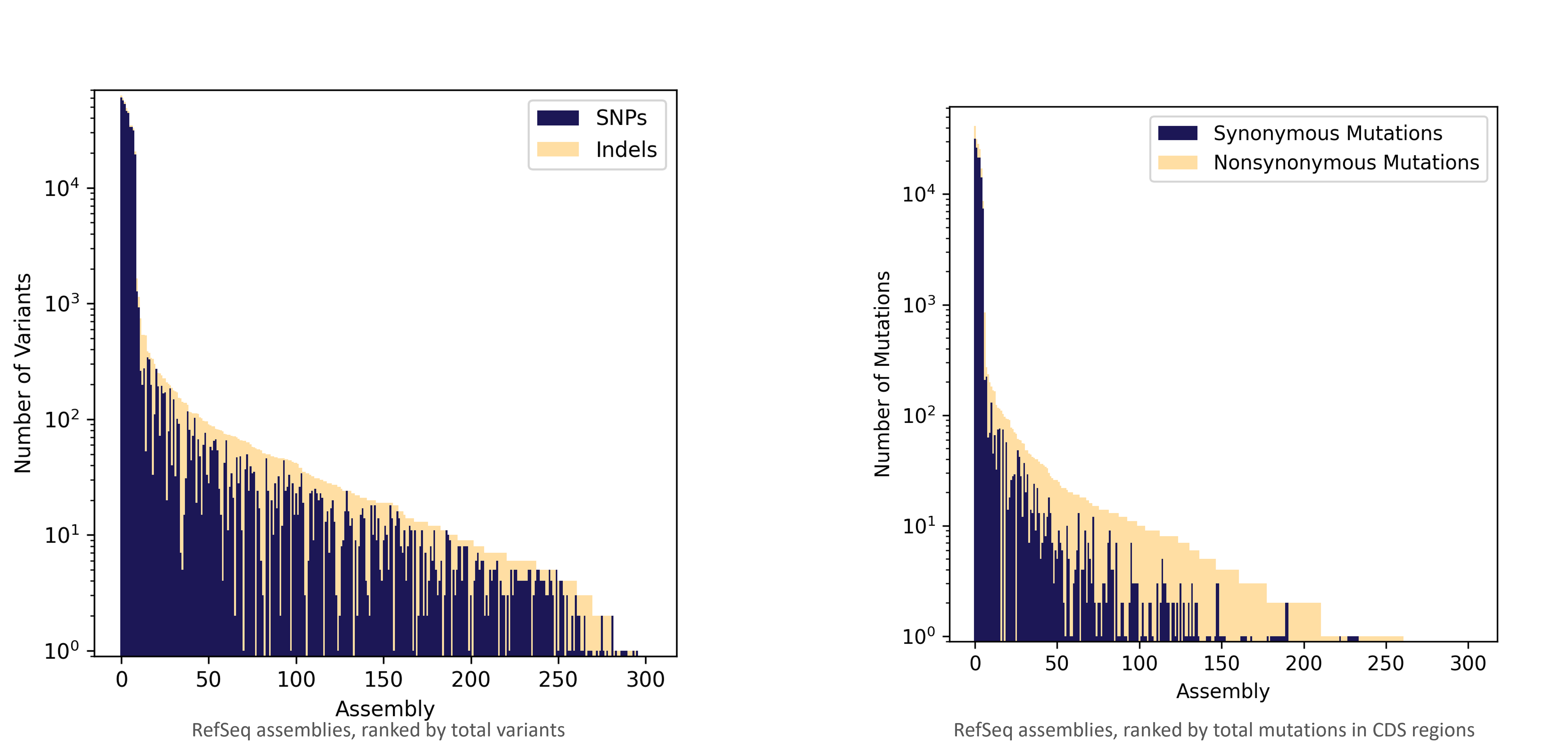

Supplement: FIG S4 [file msphere.00077-22-s0004.tif]

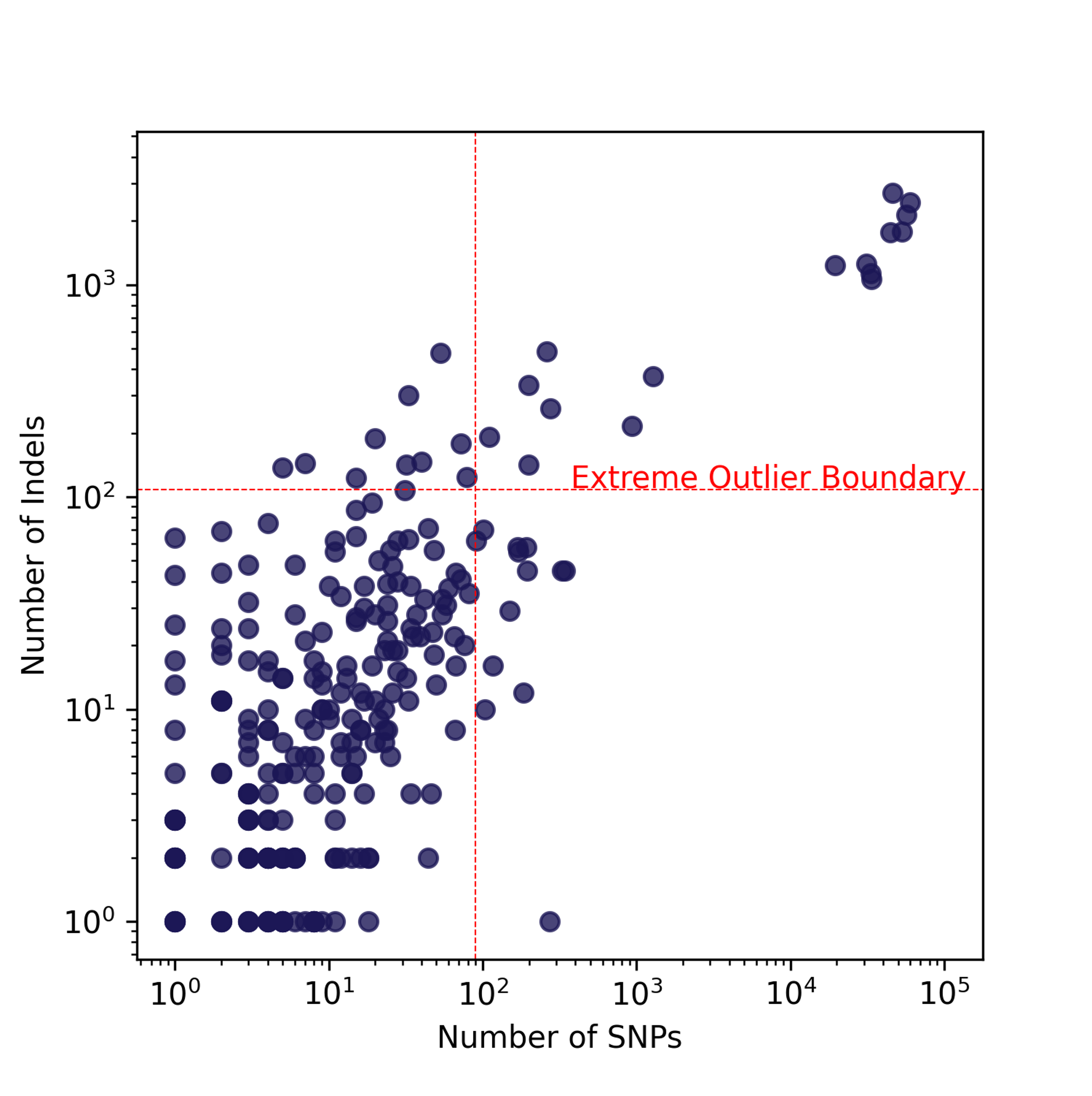

Supplement: FIG S3 [file msphere.00077-22-s0003.tif]
